# Keto Analogues in Patients with Chronic Kidney Disease with or Without Kidney Transplantation

**DOI:** 10.3390/nu16234001

**Published:** 2024-11-22

**Authors:** Patrícia Kleinová, Tímea Blichová, Karol Graňák, Andrej Kollár, Matej Vnučák, Ivana Dedinská

**Affiliations:** 1 Transplant-Nephrology Department, University Hospital Martin, Kollárova 2, 036 01 Martin, Slovakia; kleinova.pata@gmail.com (P.K.); granak.k@gmail.com (K.G.); andrej.kollar.01@gmail.com (A.K.); vnucak.matej@gmail.com (M.V.); ivana.dedinska@uniba.sk (I.D.); 2 Department of Internal Medicine I, Jessenius Medical Faculty, Comenius University, 036 01 Martin, Slovakia

**Keywords:** keto analogues, kidney transplant recipients, low-protein diet, side effects

## Abstract

***Background:*** Keto analogues in combination with a (very) low-protein diet significantly reduces the progression to end-stage kidney disease. The question of their benefit and safety for kidney transplant recipients remains. This study aimed to show the renoprotective effect and safety of the use of this method in patients with chronic kidney disease and a kidney transplantation. ***Materials:*** This was a retrospective monocentric study conducted by the transplant nephrology department in Martin, in which patients with chronic kidney disease, with or without kidney transplant therapy, who received a low-protein diet and supplementation with keto analogues were included (*n* = 59). The changes in their glomerular filtration rate, proteinemia, calcaemia, weight, and glycaemia and the side effects associated with a low-protein diet and keto analogue use were studied in the patients with chronic kidney disease with or without kidney transplantation. ***Results:*** The kidney transplant recipients had a significantly more advanced stage of chronic kidney disease (*p* = 0.0001) than the non-transplanted patients at the time of the prescription of the keto analogues (*p* = 0.0001). Furthermore, the kidney transplant recipients had a significantly longer follow-up period (*p* = 0.0001), with a difference of 27 months within subgroups. During the observed period, we recorded a decrease in glomerular filtration, but without statistical significance. In our group, we did not confirm a significant occurrence of adverse effects associated with a low-protein diet and keto analogues. ***Conclusion:*** Keto analogues reduce the progression of chronic kidney disease and stabilise glomerular filtration in patients with chronic kidney disease. Based on our analysis, treatment with keto analogues is effective and safe for kidney transplant recipients after kidney transplantation.

## 1. Introduction

Chronic kidney disease (CKD) is a disease whose prevalence (13.4% of the adult population) increases with age. Its incidence is projected to increase annually, and it is expected to represent the fifth leading cause of death in adults by 2040 [1,2,3]. However, even state-of-the-art approaches of contemporary medicine cannot provide us with a medical treatment that can cure end-stage renal disease. For this reason, as the number of patients with CKD increases, the number of patients requiring some form of renal function replacement with a predominance of dialysis is increasing in direct proportion. Compared to the general population, dialysis patients are at a higher risk of developing cardiovascular disease (CVD), which is associated with significant morbidity and mortality. According to the results of an analysis by Bello et al. in 2022, in adults aged 40–44 years, the difference in life expectancy between the general population and patients enrolled in a regular dialysis program was 20–25 years. However, the life expectancy was also 2–5 years shorter for patients on dialysis in the 80–84 age group compared to their peers [4]. Cardiovascular risk is inversely correlated with the estimated glomerular filtration rate (eGFR) and proteinuria [3].

The need to maximize non-pharmacological and pharmacological approaches to slow the progression of CKD to end-stage kidney disease (ESKD) was also highlighted by the latest Kidney Disease Improving Global Outcome (KDIGO) 2024 recommendations. First-line pharmacotherapy includes sodium–glucose cotransporter 2 inhibitors, renin–angiotensin system inhibitors, and a hypolipidemic agent. In addition to pharmacotherapy, lifestyle plays a crucial role, including a healthy diet, physical activity, the non-use of tobacco products, and weight management [5]. In patients with CKD, moderate-intensity physical activity (brisk walking, gardening, washing windows, light cycling, …) of at least 150 min per week is recommended cumulatively, taking into account the patient’s age and comorbidities [5]. A diet similar to the dietary approaches to stop hypertension is recommended in patients with CKD. This is a plant-based diet that is low in animal products and sodium and that restricts the consumption of ultra-processed foods (sweetened beverages, frozen meals, fast foods) whose nutritional value is low. The purpose of this plant-based dietary approach is to support the gut microbiome in favour of commensal bacteria, with a reduction in inflammation and the production of uremic toxins. A plant-based diet (Mediterranean, vegetarian, or vegan) containing unprocessed protein slows the decline in the eGFR and reduces proteinuria and metabolic acidosis [5].

In the context of slowing the progression of CKD, a low-protein diet is essential. The purpose of this diet is to reduce the intake of protein (especially animal protein), which, unlike carbohydrates and fats, does not build up stores in the body and is catabolized into waste products—urea and uremic toxins. With the progression of CKD, the elimination of nitrogenous metabolites is impaired, and their subsequent accumulation, the development of metabolic acidosis, and the impairment of the function of other organs occurs. High serum urea concentrations are associated with the formation of reactive oxygen species, leading to oxidative stress, endothelial dysfunction, and the progression of CVD. An increased protein intake also increases the intraglomerular pressure, with subsequent glomerular hyperfiltration and the development of glomerulosclerosis and tubulointerstitial damage [6].

According to the KDIGO recommendations, a low-protein diet (LPD) is defined as a protein intake of 0.6–0.8 g/kg body weight per day, and is recommended for metabolically stable patients with CKD G3–G5 without diabetes mellitus and without nephrotic syndrome. In patients with diabetic kidney disease, a low-protein diet at the upper limit of normal (0.8 g/kg body weight/day) is suggested [7]. In patients who are willing and able, a very low-protein diet (VLPD) with an intake of 0.3–0.4 g/body weight/day can be prescribed under strict clinical supervision with the supplementation of essential amino acids via keto analogues (KAs) [6].

Keto analogues are analogues of essential deaminated amino acids, wherein an amino group is replaced by a keto group in the amino acid molecule. KAs are converted to branched-chain amino acids in the urea cycle without nitrogen formation. The supplementation of KAs in an LPD or a VLPD provides a substrate for protein synthesis to prevent muscle cachexia. At the same time, nitrogen uptake is eliminated with the formation of toxic nitrogen products [8]. Ketosteril^®^, one of the keto analogues, can be prescribed to patients with CKD with an estimated glomerular filtration rate (eGFR) < 25 mL/min/1.73 m^2^ and who are concomitantly taking an LPD or a VLPD. The contraindications to using keto analogues are an impaired amino acid metabolism and hypercalcaemia, due to the presence of 50 mg of calcium in the keto analogue tablet [9]. There are no precise statuary recommendations for patients after a kidney transplantation and prescription; however, according to the KDIGO, we classify patients after a kidney transplantation as having chronic kidney disease [6].

In addition to using KA, the prevention of malnutrition involves maintaining an adequate caloric intake (by increasing the intake of polyunsaturated fatty acids) and ensuring that more than 60% of the protein intake has a high biological value—egg protein, beans, and soybeans [6,10].

Our study aimed to show the effect of keto analogues on eGFR and creatinemia, with a predominant focus on patients after kidney transplantation. The secondary objective was to determine the effect on glycemia, calcium, proteinemia, weight, and safety of use in CKD patients and kidney transplant recipients.

## 2. Materials and Methods

In our retrospective monocentric study, patients followed at the Transplant-nephrology department in University Hospital Martin with CKD with or without kidney transplantation who met the indication criteria for the prescription of keto analogues in 2023 were included.

### Inclusion Criteria

- Patient age > 18 years;
- Patients with chronic kidney disease KDIGO G3-5 (not received dialysis);
- Low-protein diet (0.8 g/kg body weight) or very low-protein diet (0.3–0.4 g/kg body weight);
- Absence of a history of hereditary phenylketonuria history of hereditary phenylketonuria;
- Absence of a disorder of metabolism of aminoacids.

Patients were given the keto analogue preparation (Ketosteril^®^, Ketosteril Fresenius Kabi Deutschland GmbH, Friedberg, Deuschland) at a dose of 1 tablet per 10 kg body weight (LPD patients) or 1 tablet per 5 kg body weight (VLPD patients) per day. Prior to prescribing keto analogues, patients were nutritionally educated about the general principles of the CKD diet and the principles of a low-protein diet. Each patient was given an educational brochure on the low-protein diet supplemented with example recipes.

The following parameters were ascertained in all patients studied: sex, history of diabetes mellitus, age at the time of prescription of keto analogues, and complications associated with using keto analogues. We also recorded eGFR, proteinemia, nitrogenous substances (creatinine, urea), fasting glycaemia, serum calcium concentration, and body weight at the time of prescription of keto analogues and at the time of follow-up in all participants. We divided the study sample of patients in the context of kidney transplantation (transplanted, non-transplanted) and in non-transplanted patients, we investigated the underlying cause of CKD.

The eGFR was determined using the Chronic Kidney Disease–Epidemiology Collaboration Index (CKD-EPI). We used a certified statistical program, MedCalc version 13.1.2. (MedCalc Software VAT registration number BE 0809 344,640, Member of International Association of Statistical Computing, Ostend, Belgium). Categorical variables were presented as counts and weighted percentages. Comparisons of continuous variables between groups were carried out using parametric (*t*-test) or non-parametric (Mann–Whitney) tests; associations between categorical variables were analysed using the χ-2 test and Fisher’s exact test, as appropriate. A *p*-value < 0.05 was considered statistically significant.

## 3. Results

A total of 59 adult (caucasian) patients (male: *n* = 35; 59.3%) with a mean age of 52 years were included in the study. The mean dose of keto analogues was nine tablets per day. Follow-up parameters and characteristics of the cohort with a mean follow-up of 25 months are shown in Table 1.

**Figure 1:**
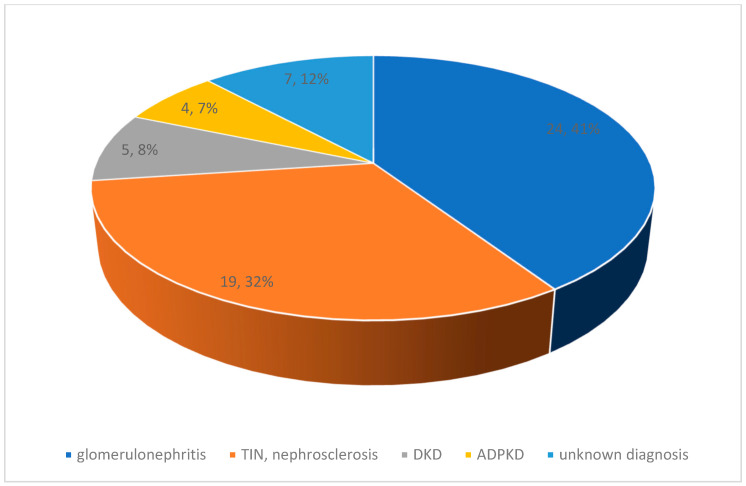
The distribution of the cohort patients according to CKD aetiology. ADPKD—autosomal dominant polycystic kidney disease, DKD—diabetic kidney disease, TIN—tubulointersticial glomerulonephritis.

**Figure 2:**
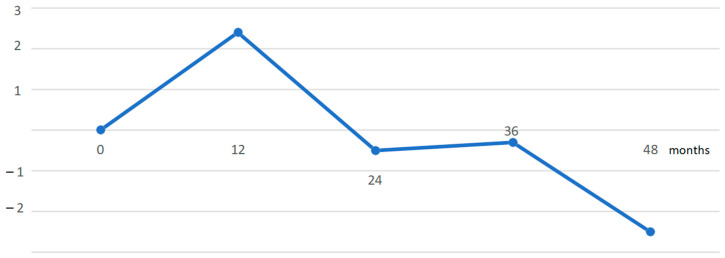
Development of eGFR during followed period in the cohort. eGFR—estimated glomerular filtration.

**Figure 3:**
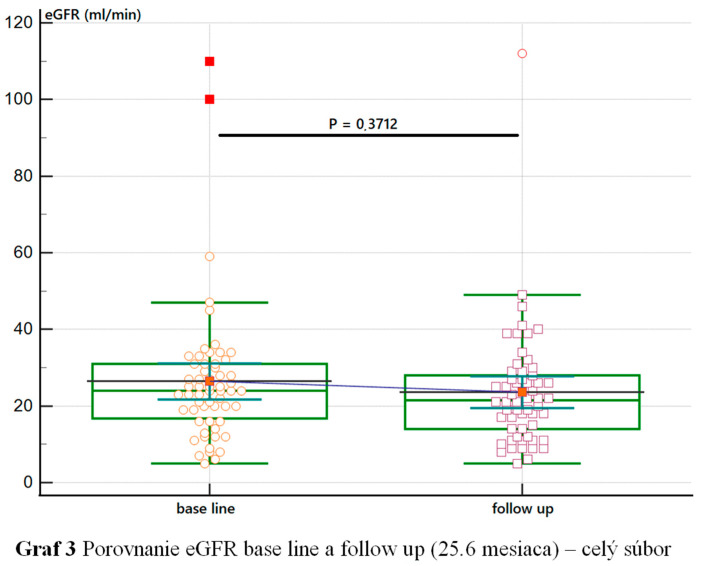
Development of eGFR in the cohort. eGFR—estimated glomerular filtration rate.

The entire cohort of patients was stratified based on a primary diagnosis of CKD with a predominance of glomerulonephritis (*n* = 24; 41%) followed by a diagnosis of tubulointerstitial nephritis (*n* = 19; 32%) (Figure 1). In the patient cohort, we observed an increase in eGFR during the first 12 months of follow-up compared with the value at the time of ketone analogue prescription. Patients with >24 months of follow-up had a decrease in eGFR in our cohort without reaching statistical significance. Patients on KA > 36 months experienced stabilization of eGFR, but patients with >48 months follow-up experienced a renewed decline in eGFR, but without reaching statistical significance and without the need for renal replacement therapy (Figure 2 and Figure 3). We did not observe any changes in the dynamics of creatinemia, glycaemia, calcaemia and weight during a mean follow-up period of 25 months. At the time of follow-up, there was only a discrete decrease in serum protein concentration compared to the value at the time of Ketosteril^®^ prescription.

**Figure 4:**
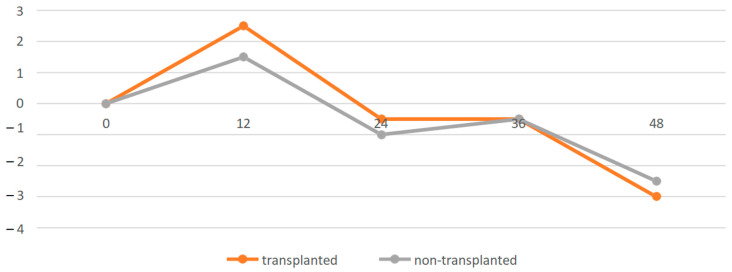
The change of eGFR during follow-up—transplanted and non-transplanted patients. eGFR—estimated glomerular filtration rate.

The sample of patients was divided into two groups regarding kidney transplantation, transplanted (*n* = 27; 45.7%) and non-transplanted (*n* = 32; 54.3%), with equal representation in both groups (Table 2). Kidney transplant recipients had a significantly higher CKD stage (*p* = 0.0001) than non-transplanted patients at the time of keto analogues prescription. In addition, transplanted patients had a significantly more extended follow-up period (*p* = 0.0001), and an average difference of up to 27 months across subgroups. Importantly, in both follow-up groups, there was only a slight decrease in eGFR without statistical significance during the entire follow-up period, up to 39 months in transplanted patients (Figure 4). Similarly, any significant changes were not detected in fasting glycaemia, serum calcium concentration, total protein or body weight within the subgroups using keto analogues. In non-transplanted patients, we focused on the aetiology of CKD, where the result replicated the representation of the underlying diagnosis in the entire cohort: patients with glomerulonephritis (*n* = 19; 59%) and tubulointerstitial nephritis (*n* = 8; 25%) dominated. Notably, during the keto analogue treatment, no hypercalcaemia, gastrointestinal distress, or other adverse effects associated with keto analogue use were confirmed in the study cohort.

## 4. Discussion

Our analysis confirmed the effect of keto analogues on slowing the progression of CKD. However, the advanced effect associated with keto analogues used was not confirmed even in kidney transplant recipients.

In our analysis, the average dosage of keto analogues was nine pills per day. However, there needs to be greater consensus in the literature on the dosage of KA in the context of individual low-protein diets. An analysis by Garneat L. et al. performed in patients with CKD showed that the dosage of keto analogues in patients with LPD was the same as in our group [11]. The mean daily dose of keto analogues in the 2023 study by Ariyanopparut et al. was significantly lower than the daily dose in our analysis and was 4.4 tablets [12]. In the past, KA supplementation in patients with LPD was optional, in contrast to patients with VLPD, where it was mandatory [13]. However, later studies highlighted that CKD progression was less steep in LPD patients with KA supplementation than without [14]. This was confirmed by the 2023 study mentioned above, where patients taking six or more keto analogues tablets daily were shown to have a slower progression of CKD or onset of dialysis [12].

In our group, with a mean follow-up of 25 months, an increase in glomerular filtration rate was detected during the first 12 months of keto analgesic use, which may be attributed to patients’ consistent adherence to LPD. Patients with a follow-up of >24 months had an identified decrease in eGFR, but this did not reach statistical significance. Patients with follow-up of >36 months experienced a stabilization of eGFR, followed by a re-decrease in patients taking keto analogues for >48 months, but no statistical significance was reached, and there was no need to initiate dialysis treatment. Similar results were obtained in an analysis by Garneat et al. [11]., which included diabetic kidney disease patients with LPD supplemented with KA for 12 months. In that group of patients, there was a significant increase in eGFR in the first phase of the study, followed by a decrease in glomerular filtration rate during the follow-up period, corresponding to the physiological decrease in eGFR in the population. The authors assess the initial decrease as an effect of LPD. Our results also supported the findings of a meta-analysis by Li A et al. performed with seven randomized control trials (RCTs) and one non-RCT, where LPD supplemented with keto analogues significantly delayed the progression of CKD reaching statistical significance, especially in patients with eGFR > 18 mL/min/m^2^. The authors also looked at the difference in change in eGFR in a comparison between LPD and VLPD, which showed no differences [15].

In the context of eGFR decline, two limitations were identified in our analysis—patient non-compliance to LPD and keto analogue dosing. Since it is a strict diet with many restrictions, patients’ motivation and ability to comply with its principles may broadly decline after a certain period. Some patients may find preparing meals complicated and time-consuming and therefore reach for more readily available meals in public canteens. Patients with diabetic kidney disease, whose diets already contain restrictions due to the underlying disease, represent a particular group of patients at risk from a compliance perspective. Therefore, we aim to improve doctor–patient cooperation with continuous feedback and careful evaluation of dietary change through food intake recording. Keto analogues dosing was evaluated as a reserve of our cohort to increase the daily dose of keto analogues. In patients with progressive CKD, despite a stable daily dose of 1 tablet per 10 kg of body weight per day, we suggest an increase to 1 tablet for 5 kg of body weight per day, which corresponds to the dose recommended for VLPD, which in reality is very difficult to achieve by patients.

Both renal transplant patients and non-transplant patients were included in our analysis, and their representation in the cohort was comparable. In transplanted patients, we started LPD and prescription of keto analogues later—at a later stage of CKD. The reason for this is the initial focus on diagnosing and treating the immunological cause of impaired renal allograft function. Follow-up in transplanted patients was significantly longer in our series than in non-transplanted patients. This is due to a change in the organization of our department, whereby, in 2022, the dominant part of our patients will be renal transplant patients, with a subsequent extension of the spectrum of patients to include non-transplant patients. In both follow-up groups, the eGFR change curve during follow-up followed the eGFR change curve of the whole cohort without achieving a statistically significant decrease in eGFR. Minimal data on the prescription of keto analogues in patients after kidney transplantation are present in the available literature. Studies have predominantly focused on patients with CKD before kidney transplantation. However, in the context of similar outcomes in both groups of our cohort, we assess the prescription of keto analogues as safe and significantly beneficial for the patient. In the available literature, there are no data on the interaction of immunosuppressive therapies (calcineurin inhibitors, corticosteroids, mycophenolic acid) especially and keto analogues. In our study, this interaction was not confirmed.

In the entire monitored group and within the subgroups (transplanted, non-transplanted patients), we also focused on changes in calcium, fasting plasma glucose, proteinemia and weight in the context of LPD and the use of keto analogues during the monitored period. In the entire group, hypercalcemia was not detected as the most frequent adverse effect associated with the use of keto analogues. A study by Jiang Z. et al. also did not find changes in serum calcium levels, but in the meta-analysis, Li A. et al. showed higher values of calcium in patients using KA with worse renal function (eGFR < 18 mL/min/m^2^) [15,16]. Low-protein diets are associated with the risk of malnutrition. In our group of patients, we also focused on changes in the nutritional parameters of proteinemia and body weight, which did not reach a statistically significant change. Our results confirmed the conclusions of studies where no decrease in albuminemia or body weight was detected in patients taking KA compared to the control group, even in patients with VLPD [15,17].

In studies conducted on patients with diabetic kidney disease, a statistically significant decrease in fasting glucose was noted with LPD and keto-analogues supplementation. It is believed that the cause is an improvement in insulin resistance with a decrease in nitrogenous substances, and because keto analogues contain nitrogen-free amino acids, gluconeogenesis from amino acids is reduced. However, our analysis did not show a change in fasting glucose, but the reason may be the low number of patients with diabetes mellitus included in our study (*n* = 14) [18].

A limitation of our study was the absence of a control group; however, in the setting of our study, all patients meeting the study criteria are consistently prescribed Ketosteril as part of their treatment protocol, precluding the formation of a proper control group. When patients do not take Ketosteril, it is usually due to specific, individual limitations (such as gastrointestinal problems or difficulty swallowing large tablets), which are relatively rare in our patient population. As a result, this control subgroup would be small and heterogeneous, making it statistically and clinically incomparable to the treatment group.

## 5. Conclusions

A low-protein diet supplemented with keto analogues provides significant benefits for the CKD patient in the context of slowing the progression to end-stage chronic kidney disease with the need for dialysis-dominant renal function replacement. As is generally known, by enrolling a patient in a regular dialysis program, we increase their morbidity and mortality. Our analysis, as one of the few, confirmed the named effect of keto analogues also in patients after kidney transplantation, when by extending the life of the graft, we can gain time to prepare for the implementation of secondary or tertiary kidney transplantation with a focus mainly on a pre-emptive approach. The adverse effects associated with keto analogues and LPD, such as hypercalcemia and protein malnutrition were not confirmed, which gives keto analogues an excellent safety profile even in kidney transplant recipients.

## Figures and Tables

**Table 1:** The basic characteristics of the cohort.

age (years) ± SD	52.1 ± 14.9
gender—men (%)	59.3 (*n* = 35)
kidney transplantation (%)	45.8 (*n* = 27)
diabetes mellitus (%)	23.7 (*n* = 14)
body weight baseline (kg) ± SD	82.7 ±17.9
fasting glycaemia baseline (mmol/L) ± SD	5.2 ± 1.1
serum proteins baseline (g/L) ± SD	66.2 ± 6.7
creatinine baseline (μmol/L) ± SD	286 ± 154
urea baseline (mmol/L) ± SD	19 ± 8.9
eGFR baseline (ml/min) ± SD	26.4 ± 18.1
calcium baseline (mmol/L) ± SD	2.4 ± 0.2
CKD stage baseline (KDIGO) ± SD, median	4.2 ± 0.9, median 5
body weight follow-up (kg) ± SD	82.6 ± 17.7
fasting glycaemia follow-up (mmol/L) ± SD	5.5 ± 1.6
serum proteins follow-up (g/L) ± SD	65.8 ± 7.4
creatinine follow-up (μmol/L) ± SD	303 ± 161
urea follow-up (mmol/L) ± SD	17.8 ± 7.6
eGFR follow-up (ml/min) ± SD	23.6 ± 15.7
calcium follow-up (mmol/L) ± SD	2.4 ± 0.2
follow-up (months) ± SD, median	25.6 ± 25.1, median 13
average dose of keto analogues (tbl/day) ± SD, median	9 ± 2, median 9

CKD—chronic kidney disease, eGFR—estimated glomerular filtration rate according CKD-EPI (Chronic Kidney Disease Epidemiology Collaboration), KDIGO–The Kidney Disease: Improving Global Outcomes, SD—standard deviation.

**Table 2:** Comparison of monitored parameters in transplanted and non-transplanted patients.

	Transplanted Patients *n* = 27	Non-Transplanted Patients *n* = 32	*p* Value
age (years) ± SD	52.7 ± 14.9	51.7 ± 15.1	0.7997
gender—men (%)	51.9 (*n* = 14)	65.6 (*n* = 21)	0.4241
diabetes mellitus (%)	25.9 (*n* = 7)	21.9 (*n* = 7)	0.8657
body weight baseline (kg) ± SD	83.1 ± 20.3	82.5 ± 16	0.8994
glycaemia baseline (mmol/L) ± SD	5.3 ± 1	5.2 ± 1.2	0.7323
serum proteins baseline (g/L) ± SD	67.6 ± 5.9	65 ± 7.2	0.1395
creatinine baseline (μmol/L) ± SD	292 ± 152	281 ± 158	0.7873
urea baseline (mmol/L) ± SD	19.9 ± 8.6	18.3 ± 9.2	0.4958
eGFR baseline (ml/min) ± SD	24.5 ± 11.6	28 ± 22	0.4603
calcium baseline (mmol/L) ± SD	2.4 ± 1.2	2.3 ± 1.2	0.7510
CKD stage baseline (KDIGO) ± SD, median	4.7 ± 0.6, median 5	3.8 ± 1, median 4	0.0001
body weight follow-up (kg) ± SD	82.9 ± 19.6	82.4 ± 16.3	0.9152
glycaemia follow-up (mmol/L) ± SD	5.7 ± 1.8	5.3 ± 1.3	0.3270
serum proteins follow-up (g/L) ± SD	65.9 ± 7.6	65.7 ± 7.4	0.9190
creatinine follow-up (μmol/L) ± SD	302 ± 154	304 ± 170	0.9627
urea follow-up (mmol/L) ± SD	18.2 ± 7.5	17.5 ± 7.7	0.7261
eGFR follow-up (ml/min) ± SD	21.4 ± 10	25.5 ± 19.4	0.3255
calcium follow-up (mmol/L) ± SD	2.5 ± 1.2	2.4 ±1.1	0.7398
follow-up (months) ± SD, median	39.6 ± 29.2, median 42	12.5 ± 12, median 11	0.0001
average dose of ketoanalogues (tbl/day)	9 ± 2, median 9	9.2 ± 2, median 9	0.7034

CKD—chronic kidney disease, eGFR—estimated glomerular filtration rate according CKD-EPI (Chronic Kidney Disease Epidemiology Collaboration), KDIGO–The Kidney Disease: Improving Global Outcomes, SD—standard deviation.

## Data Availability

The original contributions presented in this study are included in this article; further inquiries can be directed to the corresponding author.
